# Examination of Ankle Trauma in United States Military Members: A Scoping Review

**DOI:** 10.7759/cureus.32175

**Published:** 2022-12-04

**Authors:** Himbert J Sinopoli, Audrey A Vasauskas

**Affiliations:** 1 College of Medicine, Alabama College of Osteopathic Medicine, Dothan, USA; 2 Institutional Effectiveness, Alabama College of Osteopathic Medicine, Dothan, USA

**Keywords:** ankle sprains, military personnel, return to elective service, ankle injuries, military trauma

## Abstract

Military members, along with their injuries and expectations to return to duty, vary significantly from the general population. The purpose of this scoping review is to examine current literature pertaining to ankle injuries in United States military members and identify the research gaps. A scoping review was carried out with the PRISMA-ScR guidelines. A systematic search was utilized on three databases: PubMed, AMED, and Cochrane Library. The papers included were those of ankle injuries in the military. Exclusionary criteria included papers that did not have quantitative data, no full paper availability and non-US military. One hundred and fifty articles were screened, with nine meeting the inclusion criteria. Almost all of these were cohort by design. The focus was on a variety of tests for returning to active duty and the risks associated with individual factors for each service member. The study identified a lack of randomized control trials, underrepresentation of vulnerable subgroups within the US military, and no single test for ankle mobility and strength to return to duty. Prioritizing these deficiencies may help to save time and money within the US military healthcare system.

## Introduction and background

The military healthcare system faces its own unique challenges while serving a subset of the United States population. More than 70% of each military branch’s enlisted soldiers are under 24 years [1], while the average US total population under 24 is 43.3% [2]. Demands for military occupation as well create a focus on the musculoskeletal system is not seen at the same rate in the general population. These large ground impact forces are the main culprit of lower-body injury seen in military personnel [3].

One delicate and regularly injured joint of the lower body is the ankle. Ankle fractures are fairly common in the civilian population, with an incidence of 187 per 100,000 per year. However, the most common demographic associated with ankle fractures are elderly women [4].

With differing demographics and occupations, the US military faces its own demands in healthcare. Injuries are no abnormality within the US military, with a rate of more than 1800 cases per 1000 Army soldiers per year [5]. From physical requirements such as running and parachuting-oftentimes on less than ideal surfaces-ankle injuries are more common than in the general population [6]. This scoping review aims to establish a current base of literature pertaining to US military ankle injury and identify the gaps for further research.

## Review

Materials and Methods

The scoping review adhered to the PRISMA-ScR [7] guidelines. Only articles in English and published within the last five years were included. Exclusionary criteria included no quantitative data, no full paper available, and non-US military. Three databases were searched: PubMed, AMED, and the Cochrane library. MeSH terms were used in these searches. The terms searched were: (ankle injury OR ankle fracture) AND (military personnel OR military health). Zotero was utilized to manage the articles between the three databases. The screening was done by the reviewer to eliminate papers first by title and then by abstract. Finally, using the after mentioned exclusionary criteria, articles were selected for inclusion in the scoping review.

Results

**Figure 1:**
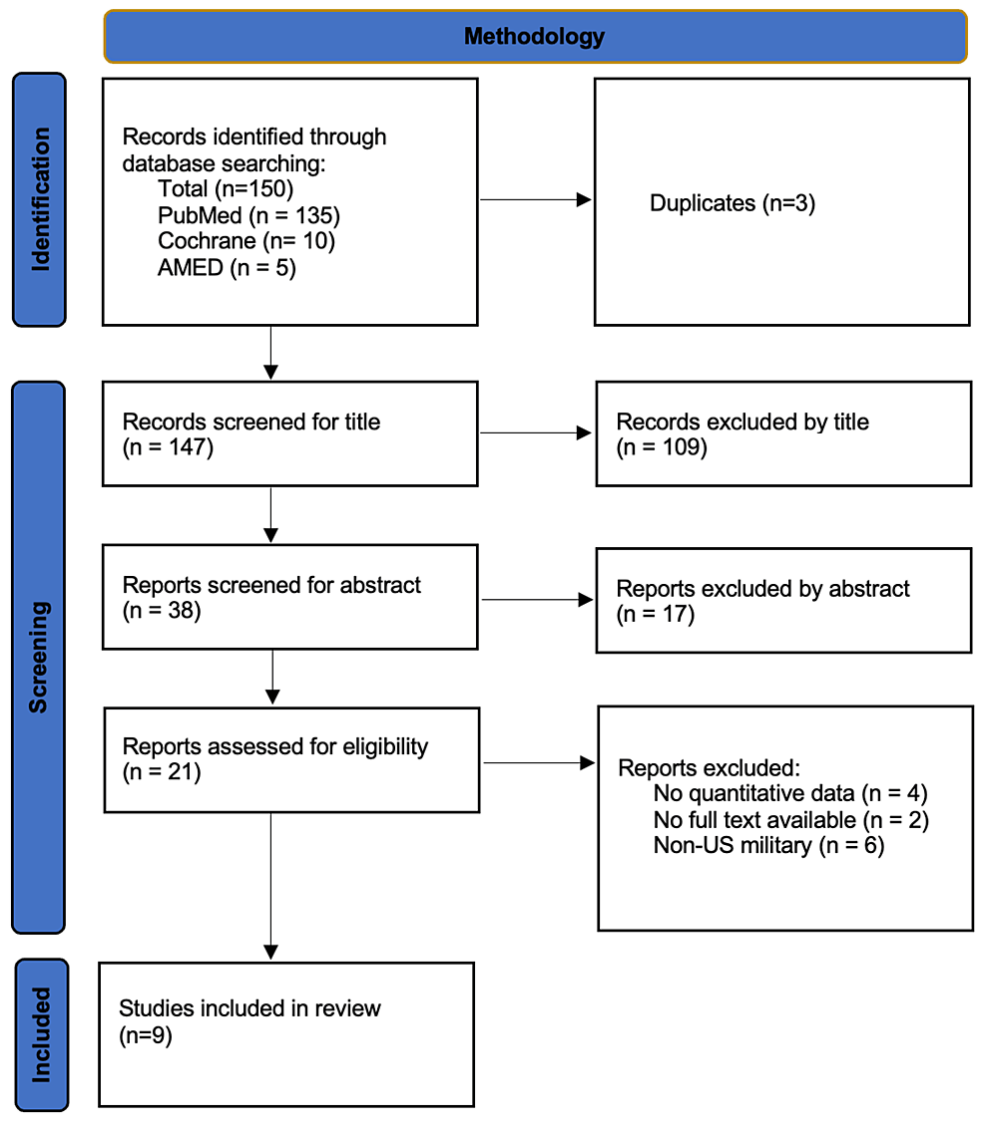
PRIMSA flow diagram detailing search strategy

A summary of the search strategy and inclusions is shown in Figure 1. One hundred and fifty articles were selected for screening, with 135 from PubMed, 10 from Cochrane, and five from AMED. After removing duplicates, an additional 126 articles were removed via title or abstract. The final inclusion criteria eliminated 12 more articles. In total, 19 articles have been included in the scoping review.

Studies included in the scoping review are found in Table 1 with their associated findings. A majority of the articles included are designed as cohort studies, 88.8% (8/9). The other, a case report, included a population with special forces background. This population accounted for one-third of the studies. Multiple studies used different tests to determine rehabilitation from injury. These tests range include: single leg landing [8], asymmetry [9], risk factors [10], Functional Movement Screen (FMS) [11], the American Foot and Ankle Score Outcome (AOFAS), Single Assessment Numeric Evaluation (SANE) [12], and procedure and delaying outcomes [13-15]. 

**Table 1:** Included articles with aim, design type, population, and findings US: United States, SANE: Single Assessment Numeric Evaluation

Author	Aim of Research	Design	Sample and Population	Findings
Bansbach et al, 2017 [8]	To evaluate dynamic stability and landing kinematics in those with ankle injuries	Cohort study	55 Special Forces Operators	One year after injury, there was no significant difference in stability and kinematics between the injured and uninjured groups
Eagle et al, 2019 [9]	To determine the role of asymmetry in the role of the ankle injury	Prospective cohort over one year	140 male US Air Force Special Forces	Asymmetry was unable to significantly predict ankle injuries as an independent variable
Fraser et al, 2021 [10]	To assess lateral ankle sprain rates across the US military to account for sex and occupation	Retrospective cohort	360,256 members of the US military with code for lateral ankle sprain	Sex and occupation were significant in the relative risk of lateral ankle sprain
Gutschick and Lazicki, 2020 [11]	To detail a system to return to duty following an ankle fracture	Case report	34-year-old male Special Forces Operator	Following the system, the patient has a functional fitness score that was within the standard deviation for class averages
Johnson et al, 2019 [12]	To evaluate outcomes after ankle fracture	Retrospective cohort	43 active-duty service members	Higher SANE scores were associated with a higher percentage of return to active duty
Junge et al, 2017 [13]	To report surgical outcomes of high-energy talus fractures	Retrospective cohort	48 US service members	Talus fractures from high-energy trauma were associated with more amputations and subsequent surgeries
Melton et al, 2017 [14]	To evaluate outcomes from the modified Broström procedure	Retrospective cohort	127 US service members	The modified Broström procedure allowed a majority of members to stay in the service
Rhon et al, 2021 [15]	To determine the significance of starting rehabilitation early and future injury	Retrospective cohort	22,475 US service members	Delayed rehabilitation resulted in a higher number of recurrent injuries
Rhon et al, 2021 [16]	To examine the burden of ankle injuries on the US military health system	Retrospective cohort	30,910 US service members	Ankle sprains are commonly associated with future fractures and high-cost downstream

Discussion

With less than one-half of a percent of the United States total population [1], it is to be expected that the final number of journals included was not immense. Nonetheless, the role of the military and its personnel still require a large number of resources for its medicine.

A critical source for many of the articles was the databases for the United States military. These include the US military Health System Data Repository (MHR) [15-16], the Defense Medical Epidemiology Database (DMED) [10], and the Department of Defense Trauma Registry (DODTR) [13]. These convenient databases lend themselves to massive amounts of information within military personnel. It should be no surprise then that retrospective cohort studies were vastly overrepresented in this field of study. Other cohort studies included either single military units [9] or took individual hospital databases [12,14]. Notably absent from the design are randomized control trials (RCT). RCTs are the gold standard when it comes to research design evidence-based medicine for their lack of bias and minimization of errors [17].

Another potential gap in the literature is the lack of representation in non-special operations units. As previously mentioned, this group accounts for one-third of the articles. This is despite that special operators have the lowest relative risk for lateral ankle sprains among enlisted personnel. On the other hand, artillery and gunnery positions had no specific research while also having the highest risk [10]. Within the highly specialized military are even more specialized roles that require their own protocol to prevent, care for, and rehabilitate the injury.

A final gap in the literature is the lack of reliable and uniform testing before returning to duty. This is imperative in a career that requires the use of physical activities such as running in every occupation in the form of physical fitness tests [12]. These tests range from asking patients subjectively on a scale of 1-100 (SANE) how the ankle feels to the movement (FMS) to tests that combine pain and movement (AOFAS). The importance of prompt and accurate testing is key to stabilizing the ankle and possibly bringing down the number of first-time ankle sprains to develop chronic ankle instability (40%) [16]. In addition to inconsistent testing leading to a premature return, delayed rehabilitation caused problems down the line as well. Delaying just a few weeks to start rehabilitation increases both the odds ratio of future injury(1.28) and additional visits needed (1.22) [15]. These numbers are telling, as the average ankle injury in the US military healthcare setting costs approximately $2,725 [16].

This study has several limitations. First, non-English articles were not included. Although the focus of the review was on the United States military, some literature created by allies at overseas bases may not have been included. A second limitation is that three databases were searched. This could lend itself that not all obtainable literature was included in the search, but those that were included went through an extensive process to be published. A final limitation is that the review was done by a single researcher, which could lend itself to bias.

## Conclusions

This scoping review aims to establish a current base of literature pertaining to US military ankle injury and identify the gaps for further research. Three gaps were identified: a lack of studies outside of cohort design, overrepresentation of special operators in ankle injury, and lack of consensus on an ankle stability test that predicts long-term health. Attempts to advance these shortcomings may help to improve the efficiency around ankle injuries as well as improve outcomes for years to come.
